# Imaging of lumpectomy surface with large field-of-view confocal laser scanning microscope for intraoperative margin assessment - POLARHIS study

**DOI:** 10.1016/j.breast.2022.10.003

**Published:** 2022-10-05

**Authors:** Mariana-Felicia Sandor, Beatrice Schwalbach, Viktoria Hofmann, Simona-Elena Istrate, Zlatna Schuller, Elena Ionescu, Sara Heimann, Moira Ragazzi, Michael P. Lux

**Affiliations:** a Department of Gynecology and Obstetrics, Women's Hospital St. Louise, Paderborn, Women′s Hospital, St. Josefs, Salzkotten, St. Vincenz-Krankenhaus GmbH, Husener Str. 81, 33098, Paderborn, Germany; b Pathology Unit, Azienda USL – IRCCS di Reggio Emilia, 42123, Reggio Emilia, Italy

**Keywords:** Breast conserving surgery, Confocal microscopy, Ductal carcinoma in-situ, Histolog scanner, Re-excision, Fresh tissue imaging

## Abstract

**Introduction:**

Breast-conserving surgery (BCS) in case of breast cancer and/or in-situ-carcinoma lesions (DCIS) intends to completely remove breast cancer while saving healthy tissue as much as possible to achieve better aesthetic and psychological outcomes for the patient. Such modality should result in postoperative tumor-free margins of the surgical resection in order to carry on with the next therapeutical steps of the patient care. However, 10–40% of patients undergo more than one procedure to achieve acceptable cancer-negative margins. A 2nd operation or further operation (re-operation) has physical, psychological, and economic consequences. It also delays the administration of adjuvant therapy, and has been associated with an elevated risk of local and distant disease relapse. In addition, a high re-operation rate can have significant economic effects - both for the service provider and for the payer. A more efficient intraoperative assessment of the margin may address these issues. Recently, a large field-of-view confocal laser scanning microscope designed to allow real-time intraoperative margin assessment has arrived on the market - the Histolog Scanner. In this paper, we present the first evaluation of lumpectomy margins assessment with this new device.

**Materials and methods:**

40 consecutive patients undergoing BCS with invasive and/or DCIS were included. The whole surface of the surgical specimens was imaged right after the operation using the Histolog Scanner (HLS). The assessment of all the specimen margins was performed intraoperatively according to the standard-of-care of the center which consists of combined ultrasound (IOUS) and/or conventional specimen radiography (CSR), and gross surgical inspection. Margin assessment on HLS images was blindly performed after the surgery by 5 surgeons and one pathologist. The capabilities to correctly determine margin status in HLS images was compared to the final histopathological assessment. Furthermore, the potential reduction of positive-margin and re-operation rates by utilization of the HLS were extrapolated.

**Results:**

The study population included 7/40 patients with DCIS (17.5%), 17/40 patients with DCIS and invasive ductal cancer (IDC NST) (42.5%), 10/40 patients with IDC NST (25%), 4/40 with invasive lobular cancer (ILC) (10%), and 1/40 patients with a mix of IDC NST, DCIS, and ILC. Clinical routine resulted in 13 patients with positive margins identified by final histopathological assessment, resulting in 12 re-operations (30% re-operation rate). Amongst these 12 patients, 10 had DCIS components involved in their margin, confirming the importance of improving the detection accuracy of this specific lesion. Surgeons, who were given a short familiarization on HLS images, and a pathologist were able to detect positive margins in 4/12 and 7/12 patients (33% and 58%), respectively, that were missed by the intraoperative standard of care. In addition, a retrospective analysis of the HLS images revealed that cancer lesions can be identified in 9/12 (75%) patients with positive margins.

**Conclusion:**

The present study presents that breast cancer can be detected by surgeons and pathologists in HLS images of lumpectomy margins leading to a potential reduction of 30% and 75% of the re-operations. The Histolog Scanner is easily inserted into the clinical workflow and has the potential to improve the intraoperative standard-of-care for the assessment of breast conserving treatments. In addition, it has the potential to increase oncological safety and cosmetics by avoiding subsequent resections and can also have a significant positive economic effect for service providers and cost bearers. The data presented in this study will have to be further confirmed in a prospective phase–III–trial.

## Highlights

• Breast cancer detection in surgical margins with confocal microscopy in operating room.
• Prospective study on 40 patients with invasive and in-situ breast carcinoma.
• DCIS & Invasive cancer identified in confocal images by breast surgeons.
• Confocal assessment of lumpectomy margins lead to a reduction of re-operations up to 75%.
• Potential relevant benefit for the patient as well as positive health economic effects.

## 1 Introduction

Breast cancer is the most common cancer in women, representing 25% of all women cancers [1] and numerous women develop a ductal carcinoma in-situ (DCIS). Breast-conserving surgery (BCS) is the preferred surgical treatment for women with early-stage breast cancer and/or DCIS [2]. This surgical approach intends to completely remove breast cancer while saving healthy tissue as much as possible to achieve better aesthetic and psychological outcomes for the patient. Such modality should result in postoperative tumor-free margin assessment of the surgical resection in order to carry on with next steps of the patient care in breast-conserving treatment (e.g. radiotherapy).

The identification of non-palpable (e.g. DCIS) and deep-seated tumor during BCS is difficult for surgeons despite preoperative localization tools such as wires or clips. The surgeon is relying on visual changes and palpation of subtle irregularities to guide him during cancer excision. In the case of findings detectable by ultrasound, the practice of ultrasound-guided surgery is increasing more and more. But the lesion must be sonographically visualized by the same experienced examiner pre- and intraoperatively in its whole extension. Moreover, adequate equipment and training of the surgeon are mandatory [3,4].

While these determinants enable bulk tumor assessment, they are of limited performance to adequately identify tumor infiltrations on lumpectomy surfaces [5]. Although there is no universally accepted definition of negative or close surgical margins, from 15 to 40% of patients undergo more than one procedure to achieve acceptable cancer-negative margins as part of breast-conserving treatment [[6], [7], [8]]. Applying a second or further surgical procedure has physical and psychological impact for the patient and is an economic burden for the healthcare system. It also delays the administration of adjuvant therapy, and has been associated with an increased risk of local and distant disease relapse [9]. A more efficient intraoperative assessment of the margins may solve or reduce these issues [10]. Today, there is a lack of studies with high levels of evidence demonstrating a significant improvement from alternative intraoperative margin methods in BCS - especially for the accompanying DCIS or the pure DCIS [11]. All the different approaches have specific advantages and limitations and to date, none has gained universal adoption: as an example, cavity shave margins may not differ from selective shave margins on re-excision rate for early stage breast cancer [12].

Confocal microscopy is known for decades in the biomedical field as an efficient modality for fresh tissue imaging. Confocal laser scanning microscopes (CLSM) have been specifically developed to adapt this technology for clinical use [[13], [14], [15]]. A medical device based on CLSM approach recently arrived on the market, Histolog Scanner, allowing fresh tissue ex-vivo imaging with very large field-of-view and sufficient resolution and speed to consider intraoperative applications. This will potentially allow real-time guidance during surgery and offer support in clinical decision making for the surgeon. As previously shown by the Mayo Clinic, a tailor made approach based on intraoperative en-face frozen sections enable high rate of positive margins detection [16]. CLSM approach shares two common features meaningful for the intraoperative margin assessment: 1) it is providing an image to visualize the totality of the tissue surface; 2) generated images are close to the gold standard used in the final assessment. When used in dermatology, it has been shown that basal cell carcinoma can be detected efficiently by dermatological surgeons using the Histolog Scanner device (HLS) [17,18]. In breast tissue, the use of confocal microscopy allows accurate detection of breast cancer by pathologists as shown in a study on breast biopsies with the HLS [19]. In addition, Chang et al. reported that breast surgeons are also able to differentiate breast cancer from healthy tissue in confocal images of fresh breast tissue [20]. A recent study has presented that pathologists are able to give diagnosis and correctly identify breast cancer in HLS images of full lumpectomy specimens [21]. So in the present study, usability, performance and potential clinical benefits of using the Histolog Scanner to determine lumpectomy margin status were assessed within a prospective phase–II–trial by a certified clinical breast cancer center (according to the criteria of the German Cancer Society) that is using specimen radiography (SR) and/or ultrasound (US) beside palpation as intraoperative standard-of-care.

## 2 Material & methods

### 2.1 Patient population

Patient recruitment was held from October 2020 until February 2021, 40 patients with breast cancer and/or DCIS treated with breast conserving surgery and without neoadjuvant treatment have been recruited in accordance with study protocol approved by the Ethics committee of the Ärztekammer Westfalen-Lippe and the WWU Münster on August 16th, 2020 (No. 2020-578-f-S) and registered on the NIH database (NCT05118568).

### 2.2 Equipment

**Fig. 1:**
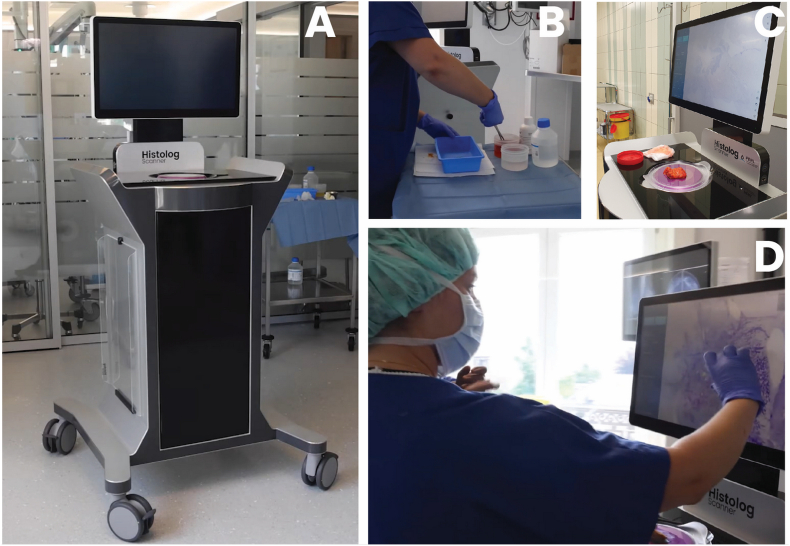
A) The Histolog Scanner; B) The specimen is stained with fluorescent dye (Histolog Dip); C) the specimen is placed on the imaging window for image acquisition; D) Image is reviewed via the touch screen of the device.

Surgical specimens were imaged with the Histolog Scanner, a CE-IVD wide field of view confocal laser scanning microscope designed for scanning large biological specimens within the operating room (here used immediately after wound closure due to the non-interventional character of the study). The scanner is a stand-alone device integrating a touch screen to operate the device and navigate into the images (Fig. 1). It requires a fluorescent dye solution (Histolog Dip) to increase contrast of the specimen and a plastic foil (Histolog Dish) to receive the specimen on the imaging window up to 8 cm diameter. Tissue fluorescence is excited by a laser at the wavelength of 488 nm and fluorescence emission is collected in the wavelength above 500 nm. The fluorescence images provide seamless images without additional post-processing, they are displayed per default with an artificial purple coloring but display in black and white mode is also accessible. The operation of the device requires simple procedures that a standard medical staff is able to realize, with a 1 h-training. The device is ready for use a few seconds after switching on without any preliminary calibration nor parameter to set by the user before imaging. A fast imaging mode provides an overview of the specimen in 5 s to check specimen positioning over the imaging window. A high-resolution mode provides images in 50 s to get access to tissue morphology details up to cell nuclei. To support image assessment, micrometric distances can be measured by user in HLS images with a digital ruler.

### 2.3 Introduction of surgeons to Histolog Scanner image content

All surgeons had no previous experience in confocal image interpretation. The first step was to make the surgeons familiar with Histolog image content of breast lumpectomies. All participating surgeons were presented supporting material showing breast cancer visualization in Histolog images. This material contains reference images of normal breast features, IDC NST, DCIS and ILC lesions with descriptions. Each surgeon reviewed the material in autonomy for 2 h distributed over 5 days at their convenience prior to the blind assessments.

### 2.4 Specimen imaging

During the surgery, the surgeon has excised the lumpectomy specimen following standard-of-care surgical practices of the certified breast cancer center which notably consists of using only cold blades and scissors to excise surgical specimen. The specimen was oriented by the surgeon with surgical threads. Right after patient closing, the specimen was gently swapped with surgical pads to remove any excess of blood. Then the specimen was stained for 10 s in the Histolog Dip fluorescent solution (SamanTree Medical, Switzerland), briefly rinsed with saline and gently swapped again with a clean surgical pad. The specimen was placed on the Histolog Scanner and images of the six specimen margins were successively acquired using Histolog Scanner in *en-face* orientation. The potential need of surgical tools such as forceps used by the surgeon to stabilize the specimen over the Histolog Scanner optical sensor was monitored together with the overall time needed for this imaging step. After Histolog imaging, surgical specimens underwent histopathology assessment following standard-of-care.

### 2.5 Breast cancer detection in histolog images

Margin status of the surgical specimen was blindly evaluated by surgeons after surgery using the Histolog Images following three predefined classes: ‘cancerous’, ‘non-cancerous’ and ‘not assessable’. Time to make this assessment was monitored.

In a second time and independently of surgeons’ assessments, a pathologist blinded to any postoperative information has reviewed the Histolog images to also determine margin status.

After this assessment, pathology reports and microscopy slides were shared with the pathologist to perform an unblinded retrospective review of Histolog images with the objective to exhaustively identify the breast cancer lesions in HLS images.

Moreover, 9 months after the initial blind assessments, 24 representative images of the positive margins were selected based on the retrospective review performed by the pathologist. 12 images were exhaustively annotated for breast cancer and/or DCIS and presented to one surgeon as a new training material. The other 12 images together with 8 cancer negative images were then presented free of any annotations to the surgeon to perform an additional qualitative blind assessment of breast cancer detection in Histolog images of lumpectomy margins.

### 2.6 Statistical analysis

The analysis was performed on all patients for each margin image (medial, lateral, cranial, caudal, ventral, dorsal), resulting in 240 images performed by HLS and analyzed by surgeons and pathologists. The performance of HLS was measured by the detection rate of positive margins identified by clinicians and with the postoperative pathology assessment as ground truth. Potential benefits on lumpectomy positive margin detection and re-operation rates were extrapolated. All continuous variables were displayed as mean and standard deviation, whereas categorical values were displayed by frequencies and percentages, when appropriate. All statistical analyses were two-tailed tests and significance was set at 5%.

## 3 Results

### 3.1 Study collective

A total of 40 patients were enrolled in the prospective phase–II–study, with a median age of 62 years (42–81 years). Patients’ lesions were non-palpable for 22 patients with an average size of 1.09 cm ± 0.84. 18 patients were presenting palpable lesions with an average size of 1.62 cm ± 0.63 (Table 1). Therefore, lesion size was slightly bigger in the palpable group, as compared to the non-palpable group (difference 0.53 cm, 95% CI 0.041–1.00; *p* = 0.03). 35% of the patients were presenting pure invasive tumors (25% of IDC NST & 10% of ILC) while 65% of the patients were presenting DCIS lesions (pure or associated to invasive tumors) (Table 1).

**Table 1:** Patient and tumor characteristics.

Parameter	Value
Age, median (STD)	62 (9.6)
Wire-guided preoperative marking	34/40 (85%)
Tumor type	
IDC NST	10/40 (25%)
ILC	4/40 (10%)
DCIS	7/40 (17.5%)
IDC NST & DCIS	17/40 (42.5%)
ILC & DCIS	1/40 (2.5%)
IDC NST & ILC & DCIS	1/40 (2.5%)
Palpable tumor	18/40 (45%)
Average size (cm)	1.62 ± 0.63
Non-palpable tumor	22/40 (55%)
Average size (cm)	1.09 ± 0.84

The type of marking was determined prior surgery in the multidisciplinary tumor board of the certified center based on the imaging and the tumor characteristics. Lumpectomies were wire-guided for 34 patients (85%) with either mammography (13 patients, including the 6 DCIS cases), ultrasound (n = 20), or a combination of the two techniques (n = 1).

### 3.2 Standard-of-care

Intraoperative margin assessment (IOA) was performed for the 40 patients with either ultrasound (US: 27 surgeries, 67.5% of the total), specimen radiography (SR: 12 surgeries, 30.3% of the total), or a combination of US and SR (1 surgery, 2.5% of the total). Intraoperative re-excisions during surgery were performed in 27.5% of the cases (n = 11/40 patients) following SoC intraoperative imaging assessment (SR or US). US assessment led to 10 intraoperative re-excisions and 1 re-excision was performed following SR assessment. Of note, US examination was performed more often in the course of the study and this technique was not significantly associated with increased intraoperative re-excisions (*p-value* = 0.17). Mean time of the intraoperative assessment was 5′46s ± 2′17s. A total of 13/40 patients (32.5%) had positive margins detected post-operatively (Table 2). Intraoperative techniques used to assess the surgical specimens of these patients were US and SR in 10/13 and 3/13 of the cases. Therefore positive margins were found in 37% (10/27) and 25% (3/12) of the surgical specimens assessed intraoperatively with US and SR, respectively. Lesions involving these positive margins were DCIS (11/13) and ILC (2/13) representing 42% (11/26) of all the patients with a tumor containing DCIS components and 33% (2/6) of all the patients with a tumor containing ILC components, respectively. No patients diagnosed with pure IDC NST had cancer-positive margin identified in the final histopathology assessment. Among the 13 patients with positive margins, 12 patients underwent re-admissions for a second breast conserving surgery resulting in a re-operation rate of 30% (12/40). 1 patient with positive margins (pure DCIS <2 mm) on the dorsal and frontal orientations did not undergo a second surgery in accordance with the guidelines of the certified center since only skin or muscle with removed fascia was shown here (Table 4).

**Table 2:** Margin assessments with standard-of-care.

Parameter	Value
Intraoperative assessment	
Ultrasounds	27/40 (67.5%)
Specimen radiography	12/40 (30%)
Ultrasounds & specimen radiography	1/40 (2.5%)
Mean time	5′46s ± 2′17s
Intraoperative re-excisions	11/40 (27.5%)
Ultrasounds	10/11 (91%)
Specimen radiography	1/11 (9%)
Postoperative margin status	
Positive margins	13/40 (32.5%)
DCIS	8/13 (61.5%)
IDC NST & DCIS	3/13 (23%)
ILC	2/13 (15.4%)

### 3.3 Margin assessment with Histolog Scanner

In addition to local techniques of imaging (US or SR) the design of the study includes the assessment of all 6 specimen sides of the excision with the HLS. This assessment is composed of an imaging step performed right after the surgery by the 5 surgeons from a breast certified center while the blind assessment on HLS images for breast cancer content is performed later on.

**Fig. 2:**
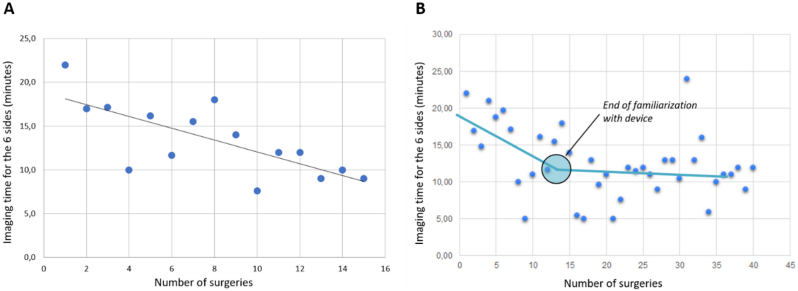
Correlation between imaging time with HLS and number of surgeries performed by A) 1 surgeon, B) all 5 surgeons.

Average time to obtain HLS images of all 6 specimen sides for each lumpectomy specimen was 12′47s ± 4′47s, which means approximately 2 min by specimen side (Table 3). Interestingly, when looking at the imaging time performed by the surgeons who performed the most image acquisition (n = 15), the time required for image acquisition decreased with the number of surgeries performed (Fig. 2A, F-statistic: 13.46 on 1 and 13 DF, p-value: 0.002837). The trend was similar when looking at all the surgeries performed in chronological order (Fig. 2). This suggests it took surgeons approximately 10 cases to get used to the specimen staining and imaging with the Histolog Scanner.

**Table 3:** Margin assessments with histolog scanner.

Parameter	Value
Use of Foerster surgical clamp	15/40 (37.5%)
Overall time	21′41 ± 5′14s
Time to image the 6 margins	12′47s ± 4′47s
Time to assess the 6 HLS images	8′54s ± 5′50s
Sensitivity (95% CI)	
Breast surgeons	30.7% (29.9%–31.5%)
Pathologist	53.8% (52.9.4%–54.7%)
Specificity (95% CI)	
Breast surgeons	85.1% (84.7%–85.6%)
Pathologist	85.2% (84.7%–85.7%)

To correctly position specimens onto the HLS, supporting tools usually available in the operating room can be used. In the course of the study, 15 specimens (37.5%) required a Foerster surgical clamp to maintain the specimen over the optical sensor (Table 3). The time to position the specimen and the usage of supporting tools were homogeneous across the different specimen sides (frontal, dorsal, lateral, medial, cranial and caudal).

Breast cancer detection in HLS images has been performed by 5 breast surgeons. Each surgeon performed on average the blind assessments on 8 patients, for the 6 specimen sides (∼48 images reviewed by each surgeon), Average time of assessing the 6 HLS margin images from one lumpectomy specimen with all 6 sides is 8′54s ± 5′50s which means approximately 1′30s per specimen side (Table 3). In a second time, a pathologist performed the blinded central review and assessment on the 40 patients (∼240 images reviewed).

**Fig. 3:**
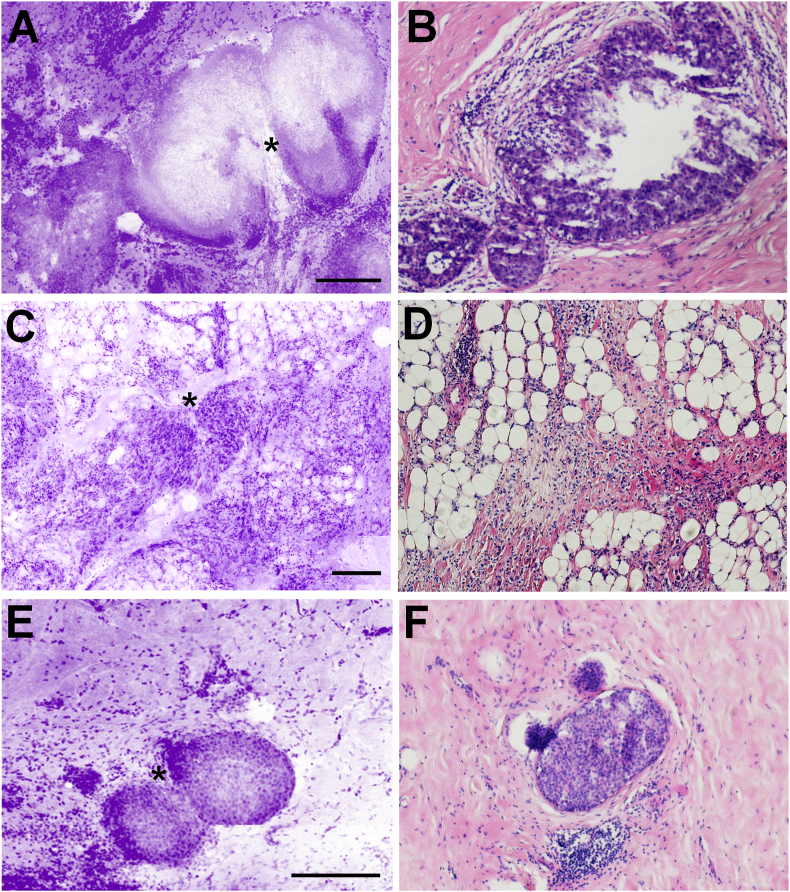
Crops of HLS images and H&E slides presenting breast cancer lesions (*) in lumpectomy margins. A&B. large DCIS; C&D. ILC; E&F. small DCIS (scale bar = 250 μm).

Breast cancer detection in HLS images was quantified with the sensitivity and specificity in comparison to postoperative histopathology reports. The overall sensitivity and specificity of the HLS assessment performed by the surgeons were 30.7% (95% CI = 29.9%–31.5%) and 85.1% (95% CI = 84.7%–85.6%), respectively (Table 3). The pathologist achieved performances significantly higher than the surgeons with a sensitivity of 53.8% (95% CI = 52.9.4%–54.7%) and a specificity of 85.2% (95% CI = 84.7%–85.7%). Physicians were able to detect both DCIS and ILC in HLS images including DCIS lesions of about 200 μm and less (Fig. 3).

Overall, among the 12 patients that had to undergo a second operation due to positive margins missed by intraoperative SoC, 4 cases with DCIS positive margins were blindly detected in HLS images by the surgeons. In addition to these four cases, pathologist was also able to detect two other DCIS cases and one case with ILC positive margin.

Following the blind assessments, a retrospective analysis of HLS images was performed by the pathologist with the support of postoperative histopathology data to check further possibilities of optimizing the detection. Positive margins from two additional DCIS patients were identified in HLS images leading to a 9/12 patients involved with DCIS or ILC. These data emphasize the potential of the HLS to improve current SoC, with a potential reduction of the positive margins achieved by surgeons and pathologists of ∼30% and ∼60%, respectively (Table 4).

**Table 4:** Breast cancer detection & re-operation rates (RoR).

Parameter	Value	Cancer Type(s)
Standard-of-Care RoR	12/40 (30%)	DCIS (10/12); ILC (2/12)
Breast cancer detection with HLS		
Surgeons	4/12 (30%)	DCIS (4/10)
Pathologist	7/12 (58.3%)	DCIS (6/10); ILC (1/2)
Pathologist (within retrospective review)	9/12 (75%)	DCIS (8/10); ILC (1/2)

Among the ten patients with positive margins involved with DCIS lesions, 40% of the in-situ components were found on ink and 60% below the surface up to 2 mm depth (Table 5).

**Table 5:** DCIS Lesions in positive margins.

Parameter	Value
DCIS localization - final assessment	
Focal DCIS	4/10 (40%)
In-depth DCIS (≤2 mm)	6/10 (60%)
DCIS localization - HLS with pathologist (retrospective review)	
Focal DCIS	3/4 (75%)
In-depth DCIS (≤2 mm)	4/6 (67%)

Interestingly, the rates of detection of these DCIS lesions by the retrospective review of the pathologist were of the same range for the margins involved on ink or below the surface suggesting that surface imaging could be also relevant for cancer lesions categorized as below the surface.

## 4 Discussion

Bringing a more accurate intraoperative assessment of lumpectomy margins may result in a higher probability of primary resection with negative surgical margins. Several techniques have been suggested in order to reduce the occurrence of cancer-positive margins and associated re-operations in BCS. These include gross examination of the lumpectomy specimen, frozen sections, touch prep analysis, intraoperative specimen radiography, and intraoperative ultrasound (US), as well as investigational tools [4,[22], [23], [24]].

Here we report the 1st evaluation of lumpectomy margins of breast cancer and/or DCIS with the Histolog Scanner, a medical device proposing the morphology assessment of large tissue specimens similar to frozen section analysis without the need for freezing or slide-preparation. The device is easy to insert in the operating room with quick preparation and imaging times that are compatible with the clinical workflow. In the present study, the assessment of the 6 sides of each lumpectomy specimen was performed by breast surgeons in approximately 20 min (12 min for imaging and 8 min for image analysis). In some cases, this time may be reduced by focusing only on the margins in which an additional shaving can be performed intraoperatively, e.g. frontal or dorsal margins may be skipped if touching the skin or if the fascia of the muscle has also been excised – especially in DCIS. This could result in a potential time reduction of 33% to end up with an overall time of assessment of ∼13 min. The device is easily operated and it may be feasible to transfer the imaging procedure to trained OR staff. If the preparation of the specimen and execution of the scan is done by an assisting surgical nurse, the surgeon could continue the operation in the meantime, for example with the sentinel node biopsy.

When assessed by the surgeons after a 2 h training, a sensitivity and specificity of 30.7% and 85.1% were found for the breast cancer detection in HLS images of lumpectomy margins. This value of sensitivity is in the same range of the values found in the literature for established imaging techniques such as specimen radiography or ultrasounds which require also an extensive training [25,26]. When assessed by a pathologist, a sensitivity and specificity of 53.8% and 85.2% were found for the breast cancer detection in HLS images of lumpectomy margins illustrating that the use of the HLS by a pathologist may already provide higher performance than established techniques from the SoC. In this evaluation, the pathologist was experienced in confocal images prior to the study but not specifically on HLS images of lumpectomy margins resulting in some uncertainty for the assessment of some images. As a further step, a retrospective review of these images was performed by the pathologist with the support of the pathology reports and microscopy slides showing an improvement of the detection rate of breast cancer up to 75% of positive margins with more experience in HLS images.

When considering the RoR, 12/40 patients had to undergo re-operations due to positive margins identified after the surgery by the histopathology assessment. Four patients with DCIS positive margins among these 12 patients (30%) were correctly identified by surgeons in HLS images showing some promise for an autonomous use of the HLS during the surgery. It is expected that their performance of breast cancer detection can be improved in a relevant way with increasing the understanding of image content. This has been qualitatively assessed 9 months after the initial blind assessments with one surgeon in which 12 representative images of the positive margins exhaustively annotated for breast cancer were presented as a supplementary training material. Then 12 cancer-positive and 8 cancer-negative additional images were blindly reviewed by the surgeon and this time, the surgeon was able to detect 100% of DCIS-positive margins and 50% of ILC-positive margins without decreasing the specificity. These promising results that will need to be confirmed in further studies are suggesting a high level of accuracy for breast cancer detection by surgeons in confocal images as previously reported [20,27]. When blindly assessed by a pathologist, 7 patients (6 with DCIS and one with ILC positive margins) were correctly identified among the 12 patients that have to undergo a second operation leading to a potential reduction of 58% of the RoR. Two additional patients with DCIS positive margins were correctly identified retrospectively with the support of postoperative data suggesting the potential of reducing the RoR of 75% with a deeper understanding of HLS image content and support of reference images. For the patients with positive margins that were not identified by the pathologist in HLS images even retrospectively (two cases with DCIS and one with ILC lesions), the use of the HLS would not bring added value to the patient that will still benefit from standard of care techniques. It can be hypothesized that the lesions were maybe not visualized in the images due to an incomplete imaging of the tissue surface, as previously reported for confocal microscopy assessments [28]. This could be corrected by increasing the experience of users to improve the flattening of the tissue surface for imaging. Performances of the pathologist for the blind identification of cancer in Histolog images of breast lumpectomies are higher than the performances of the surgeons. This could be explained by the fact that confocal images of fresh tissue are presenting tissue structures in a way that is more familiar to pathologists since they can be considered as quite similar to thicker frozen sections. Therefore, including a pathologist for the assessment with the HLS may allow to increase the benefit of using the HLS while its use in autonomy by the surgeons after a limited training may already bring some benefits for the patients.

Detecting DCIS lesions in lumpectomy margins during the surgery is one the main challenges in the BCS [29]. While the resection rate in Germany was 11.62% (n = 6.852) based on 58,967 cases in the year 2019, it was 29.17% specifically for DCIS (2.120 of 7.267 cases) [30]. This illustrates the problem of subsequent resections, especially with DCIS. These lesions are rarely palpable or presenting visual changes sufficient to guide the surgeon during the surgery [31]. When associated with microcalcifications, specimen radiography may be used to locate the lesion in the surgical specimen but this is an indirect assessment providing rough estimation of the distance to the margins and microcalcifications are not always associated with DCIS lesions [32]. To date, there are no direct techniques established in the SoC to accurately assess the presence of DCIS in lumpectomy margins during the surgery. This is illustrated by the fact that 42% of all the patients with a tumor containing DCIS component showed positive margins in the present study contributing to 85% of all the reoperations, while no positive margins were detected in patients with pure IDC NST tumor. According to the final pathology reports, approximately one third of these DCIS lesions were located on the surface of the lumpectomy margin (focal) while the remaining two third of lesions were found below the surface between 0 and 2 mm depth. Interestingly, the retrospective review of the pathologist allowed the identification of these lesions at a similar detection rate (∼75%) whatever the lesions was described to be focal or in-depth by the final pathology reports. This has to be evaluated in a larger patient population. One reason to explain this finding is that the size of the lesions identified in HLS images were sometimes very small, less than 200 μm, and this range of size may be missed by the approach of final pathology assessment that is relying on histology slides usually sampled every 2–4 mm. This is a very promising finding illustrating the potential advantage of the techniques that allow to assess the all surface of surgical margins in comparison to sampling approaches.

The concept of analysing the surface of the margins, as applied to the present study, doesn't allow to mesure the distance of cancer from the margin as it is usually reported in the final histopathology reports. This is an information that could be relevant for the intraoperative assessment of pure DCIS cases (17.5% of our study patients) and the impact of this potential limitation should be verified in further studies on larger cohort. Regarding the detection of DCIS in confocal images, it should be also considered that morphological diagnosis could be difficult in some cases. Distinguish low grade DCIS from usual ductal hyperplasia and lobular carcinoma in situ is often difficult on H&E slides and since HLS provides images with less cytological details than H&E slides, such differential diagnosis may not be rendered on HLS images. Larger prospective studies are also needed to address the impact of this potential issue.

Due to the demographic development as well as the changes in life style, cancer diseases and their cost continue to increase. In Europe, 2.45 million people develop cancer annually [33]. In total, costs of € 126 billion per year arise in the EU as a result of oncological diseases, of which € 28.4 billion are for inpatient care. Breast cancer is the most common cancer in women in Europe. Breast cancer not only represents a major diagnostic and therapeutic challenge for the various service providers, but also has significant implications for health economics due to its high incidence. Moreover, due to the increasing cost pressure, the question arises for every breast cancer center or hospital whether breast cancer therapies can be provided at a cost-covering level. Although the oncological care in certified center structures has a special focus and value in the health care system, the financing is still an often unsolved problem [34]. Several publications have already presented that care in certified breast cancer centers is not adequately remunerated and that surcharges are necessary for cost-covering work [35]. The reduction of re-operations is of particular importance, as this significantly reduces the revenue due to the merging of cases in several countries, especially in countries with the DRG (diagnosis related groups)-system. As the experience from the study described, the Histolog Scanner is able to optimize the intraoperative assessment of the margins and can potentially reduce the re-operation rate by 30–75%. As mentioned above, 6.852 re-operations were performed in Germany in 2019 [30]. By this, potentially 2056 to 5139 operations per year could be avoided alone in Germany by using the scanner - although this must of course be confirmed by a prospective interventional study. With costs of €1182.72 to €2531.44 per case with re-operation, this would have a significant health-economic effect, both for the service providers and for the payers [36].

Thus, the use of the Histolog scanner in the context of breast-conserving therapy can be of great importance for the patient, health care provider as well as for the health care system due to presented health economic aspects. The present phase–II–study is the proof-of-concept that the Histolog Scanner is able to identify positive margins for both the invasive component of breast cancer and for DCIS, and can potentially reduce the re-operation rate compared with the standard (palpation, ultrasound, X-ray). Accordingly, a prospective phase–III–study has been already planned to confirm this preliminary results on a larger cohort.

## 5 Conclusion

The present study presents that beside cancer lesions also DCIS can be detected by surgeons and pathologists in HLS images of lumpectomy margins leading to a potential reduction from 30% to 75% of the re-operations, respectively. These promising results have to be confirmed in prospective trials including more patients potentially focused on DCIS and that monitor the impact of using the HLS on the RoR. Nevertheless, this study is the proof-of-concept that the Histolog Scanner intraoperative utilization can have a relevant clinical impact and benefit for a significant proportion of the patients readmitted for re-operation, representing already a concrete added value to the current standard-of-care of breast conserving treatments. By this, in addition to optimizing oncological safety, the morbidity and the corresponding risks of a subsequent re-operation, such as complications from anesthesia, secondary bleeding, infections and reduced cosmetics, could be reduced.

## Ethical approval

This study has been performed in accordance with study protocol approved by the Ethics committee of the Ärztekammer Westfalen-Lippe and the WWU Münster on August 16th, 2020 (No. 2020-578-f-S).

## Declaration of competing interest

M.P. Lux has received honoraria from AstraZeneca, Daiichi-Sankyo Eisai, Exact Sciences, Gilead, Grünenthal, Lilly, medac, MSD, Novartis, Pfizer, pfm, PharmaMar, Pierre-Fabre, Roche, Samantree and Sysmex for advisory boards, lectures, and travel support. M.F. Sandor received travel expenses by Samantree. Other authors have no conflicts of interest.
